# Gas chromatography/mass spectrometry (GC/MS) remains a pre-eminent discovery tool in clinical steroid investigations even in the era of fast liquid chromatography tandem mass spectrometry (LC/MS/MS)^☆^

**DOI:** 10.1016/j.jsbmb.2010.04.010

**Published:** 2010-08

**Authors:** Nils Krone, Beverly A. Hughes, Gareth G. Lavery, Paul M. Stewart, Wiebke Arlt, Cedric H.L. Shackleton

**Affiliations:** Centre for Endocrinology, Diabetes and Metabolism, School for Clinical and Experimental Medicine, University of Birmingham, United Kingdom

**Keywords:** Gas chromatography/mass spectrometry, GC/MS, Tandem mass spectrometry, P450 oxidoreductase deficiency, ORD, Apparent cortisone reductase deficiency, ACRD

## Abstract

Liquid chromatography tandem mass spectrometry (LC/MS/MS) is replacing classical methods for steroid hormone analysis. It requires small sample volumes and has given rise to improved specificity and short analysis times. Its growth has been fueled by criticism of the validity of steroid analysis by older techniques, testosterone measurements being a prime example. While this approach is the gold-standard for measurement of individual steroids, and panels of such compounds, LC/MS/MS is of limited use in defining novel metabolomes. GC/MS, in contrast, is unsuited to rapid high-sensitivity analysis of specific compounds, but remains the most powerful discovery tool for defining steroid disorder metabolomes. Since the 1930s almost all inborn errors in steroidogenesis have been first defined through their urinary steroid excretion. In the last 30 years, this has been exclusively carried out by GC/MS and has defined conditions such as AME syndrome, glucocorticoid remediable aldosteronism (GRA) and Smith–Lemli–Opitz syndrome. Our recent foci have been on P450 oxidoreductase deficiency (ORD) and apparent cortisone reductase deficiency (ACRD).

In contrast to LC/MS/MS methodology, a particular benefit of GC/MS is its non-selective nature; a scanned run will contain every steroid excreted, providing an integrated picture of an individual's metabolome. The “Achilles heel” of clinical GC/MS profiling may be data presentation. There is lack of familiarity with the multiple hormone metabolites excreted and diagnostic data are difficult for endocrinologists to comprehend. While several conditions are defined by the absolute concentration of steroid metabolites, many are readily diagnosed by ratios between steroid metabolites (precursor metabolite/product metabolite). Our work has led us to develop a simplified graphical representation of quantitative urinary steroid hormone profiles and diagnostic ratios.

## 1 Introduction

Urinary steroid profiling has been a critical part of the diagnosis of disorders of steroid hormone synthesis and metabolism since the 1960s, first by thin layer chromatography, followed by gas chromatography and gas chromatography–mass spectrometry (GC/MS). However, immunoassays and related techniques have been widely used for quantification of plasma steroid hormones in routine diagnostic processes for established conditions. While such techniques have been central to our ability to diagnose clinical disorders, data are frequently compromised since the problem of cross-reactivity has never been completely solved for several analytes, thus impacting specificity. Another problem commonly occurs when low concentrations of hormones are quantified, as in pediatric patients or postmenopausal women. Furthermore, large interassay variability exists even for common measurements such as testosterone, estradiol, and progesterone [1]. These difference have been significantly lowered between laboratories using liquid chromatography tandem mass spectrometry (LC/MS/MS) [1]. The latest generation of GC/MS and LC/MS/MS techniques are superior to immunoassays regarding specificity and limits of quantification, providing a good linearity even down to low concentrations. Since LC/MS/MS high-sensitivity and high-specificity measurement can be allied with high throughput, many diagnostic laboratories are adopting this new technology. While LC/MS/MS will be the mainstream technology in steroid hormone analysis in the near future, it is not without its limitations, with one drawback being that analyte measurements are always targeted, even when panels of steroids are measured in single runs.

**Fig. 1:**
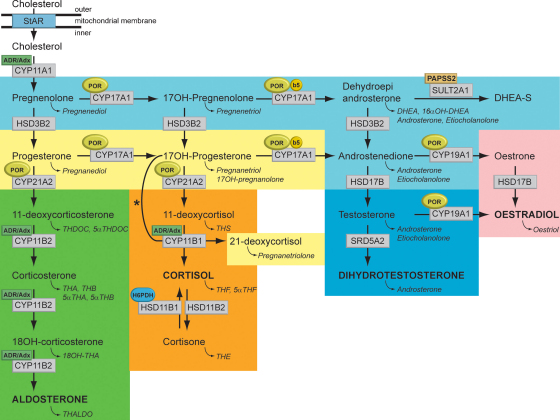
Synthesis and metabolism of hormonal steroids. This figure illustrates the formation of the major hormone classes from cholesterol. Steroid names in conventional script are steroid hormones and precursors; those in italics are urinary metabolites of the aforementioned. The major transformative enzymes are in rectangular boxes, the cofactor (“facilitator”) enzymes in ovals. The pale blue area contains common intermediate steps; the yellow preliminary steps in glucocorticoid synthesis; the green mineralocorticoids; the orange, glucocorticoids; dark blue, androgens and pink, estrogens. Mitochondrial CYP type I enzymes requiring electron transfer via adrenodoxin reductase (ADR) and adrenodoxin (Adx) CYP11A1, CYP11B1, CYP11B2, are marked with a labelled box ADR/Adx. Microsomal CYP type II enzymes receive electrons from P450 oxidoreductase (POR), CYP17A1, CYP21A2, CYP19A1, are marked by circled POR. The 17,20-lyase reaction catalyzed by CYP17A1 requires in addition to POR also cytochrome b5 indicated by a circled b5. Similarly, hexose-6-phosphate dehydrogenase (H6PDH) is the cofactor-generating enzyme for 11β-HSD1. The asterisk (*) indicates the 11-hydroxylation of 17OHP to 21-deoxycortisol in 21-hydroxylase deficiency. The conversion of androstenedione to testosterone is catalyzed by HSD17B3 in the gonad and AKR1C3 (HSD17B5) in the adrenal. StAR, steroidogenic acute regulatory protein; CYP11A1, P450 side-chain cleavage enzyme; HSD3B2, 3β-hydroxysteroid dehydrogenase type 2; CYP17A1, 17α-hydroxylase; CYP21A2, 21-hydroxylase; CYP11B1, 11β-hydroxylase; CYP11B2, aldosterone synthase; HSD17B, 17β-hydroxysteroid dehydrogenase; CYP19A1, P450 aromatase; SRD5A2, 5α-reductase type 2; SULT2A1, sulfotransferase 2A1; PAPPS2, 3′-phosphoadenosine 5′-phosphosulfate synthase 2.

The targeted analytes for radioimmunoassays (RIAs) or tandem MS analysis are selected steroid hormones and their precursors illustrated in the steroid biosynthetic pathway (Fig. 1, abbreviations listed in Table 1). Historically the analytes requested for patient studies have been kept to a minimum because of cost and lack of availability of tests for less common analytes. However, with the advent of tandem MS panels steroid hormone profiles will be routinely measured.

**Table 1:** Abbreviations of steroid hormone metabolites.

Abbreviation	Full trivial name	Metabolite of
An	Androsterone	Androstenedione, testosterone, 5α-dihydrotestosterone, DHEA
Et	Etiocholanolone	Testosterone, DHEA
DHEA (and sulfate)	Dehydroepiandrosterone	Dehydroepiandrosterone
11β-OH-An	11β-Hydroxy-androsterone	11β-OH-androstenedione, cortisol
11β-OH-Et	11β-Hydroxy-etiocholanolone	Cortisol
11-OXO-Et	11-Oxo-etiocholanolone	Cortisol
16α-DHEA	16α-OH-DHEA	Dehydroepiandrosterone, Dehydroepiandrosterone-sulfate
PD^a^	Pregnanediol^a^	Progesterone, 11-deoxycorticosterone
5PD	Pregnenediol	Pregnenolone
Pregnadienol^b^	Pregnadienol	Pregnenolone (pregnenediol)
17HP	17-OH-pregnanolone	17-OH-progesterone
3α5α17HP	3α5α-17-OH-pregnanolone	17-OH-progesterone and other fetal origin
PT	Pregnanetriol	17-OH-progesterone
5PT	5-Pregnenetriol	17-OH-pregnenolone
PTONE	Pregnanetriolone	21-Deoxycortisol
THDOC	Tetrahydrodeoxycorticosterone	11-Deoxycorticosterone
5αTHDOC	5α-Tetrahydrodeoxycorticosterone	11-Deoxycorticosterone
THS	Tetrahydro-11-deoxycortisol	11-Deoxycortisol
THA	Tetrahydro-11-dehydrocorticosterone	Corticosterone
5αTHA	5α-Tetra-11-dehydrocorticosterone	Corticosterone
THB	Tetrahydrocorticosterone	Corticosterone
5αTHB	5α-Tetrahydrocorticosterone	Corticosterone
THALDO	Tetrahydroaldosterone	Aldosterone
THE	Tetrahydrocortisone	Cortisol, cortisone
THF	Tetrahydrocortisol	Cortisol
5αTHF	5α-Tetrahydrocortisol	Cortisol
α-Cortolone	α-Cortolone	Cortisol, cortisone
β-Cortolone	β-Cortolone	Cortisol, cortisone
α-Cortol	α-Cortol	Cortisol
β-Cortol	β-Cortol	Cortisol
6β-OH-cortisol	6β-OH-cortisol	Cortisol

a When not specified the A-ring configuration is 3α,5β- in all abbreviations.

b A chemical artifact formed exclusively from 5PD (pregnenediol) disulfate.

An alternative analytical technique available for 40 years is gas chromatography mass spectrometry (GC/MS). GC/MS is used to analyse the *metabolites* of steroid hormones and their precursors (steroid metabolite abbreviations in italic script in Fig. 1). This technique can readily be utilized in comprehensive (full scan) and targeted (selected ion) mode. A “scanned” GC/MS run contains all steroids excreted and the data set can be searched for any required analyte even years after the analysis. For the last 70 years almost all disorders of steroid hormone biosynthesis and metabolism have been characterized and first named following urine steroid analysis. The most recently described examples have had their steroid phenotype established using GC/MS, emphasizing the diagnostic power and discovery potential of this technique.

Many believe tandem MS sounds the death knell of GC/MS. In contrast, while we believe that LC/MS/MS of serum steroid hormones will dominate clinical analysis, GC/MS of excreted metabolites will retain an important role. The techniques will be complementary rather than competitive. LC/MS/MS will be the method of choice to determine targeted steroids and profiles of such, whereas GC/MS will probably remain the pre-eminent tool for novel steroid metabolome discovery. GC/MS has also an additional role as a non-invasive and integrative method for known disorders of steroidogenesis, particularly useful in young infants.

One of the biggest drawbacks to the adoption of GC/MS methodology in clinical steroidology has been the presentation of data in a simple form, interpretable by endocrinologists unfamiliar with the intricacies of steroid metabolism. We present an improved visual presentation of GC/MS data to make these complex metabolomes user-friendly and easier to understand for clinicians and scientists. To illustrate this, we provide two recent examples of the use of GC/MS to biochemically define novel entities in steroid hormone biology.

## 2 Methodology utilized

The methods of sample work-up for GC/MS analysis (conjugate hydrolysis, extraction and derivatization) have been published in several papers [2], [3]. While the methodologies have been improved over the years the original conditions developed and proven for extraction, hydrolysis and derivatization documented in Shackleton's review from 1986 [4] essentially hold true. A recent paper [5] is very comprehensive as it details the selected-ion-monitoring method (SIM) for diagnosis of most steroid biosynthetic and metabolic disorders. We commonly target about 40 steroids for SIM analysis which cover all disorders of steroid synthesis and metabolism. However, we always have a back-up scanned run in case other measurements are desired in the future.

## 3 Comparison of GC/MS and LC/MS/MS for steroid analysis

A comparison of the technical features of both techniques is summarized in Table 2. LC/MS/MS has the advantage of rapid specific analysis of a limited number of compounds at high sensitivity and it is relatively easily automated. Hydrolysis of conjugates and chemical derivatization are not required. Thus, LC/MS/MS is ideally suited to routine diagnostics. Structural characterization of novel steroids is difficult by this technique and “scanning” runs of ill-defined metabolomes are difficult to obtain and interpret.

**Table 2:** Comparison of the performance of GC/MS and LC/MS/MS for steroid analysis.

Task	GC/MS	HPLC/MS
Ease of sample prep	Time-consuming	Minimal
Derivatization	Necessary	Generally not needed
Automation	Injection only	All stages.
Speed of analysis	Long	Short
Chromatographic resolution	Excellent	Poor (short run time)
Steroid conjugate detection	No	Good
Epimer separation	Good	More difficult
Specificity	Excellent	Excellent
Sensitivity 3-oxo-4-ene steroids	Moderate	Excellent
Sensitivity 3-hydroxysteroids	Good	Poor
Non-targeted steroid profiles	Good	Poor
Non-polar compounds (sterols)	Good	Poor, derivatization necessary

GC/MS has the advantage over LC/MS/MS of inherently better chromatographic resolution. LC column resolution has improved greatly recently through the use of small particle size packings (UPLC, ultraperformance LC) but is still compromised due to the need for short run times. GC/MS commonly relies on derivatization of steroids using methyloxime-trimethylsilyl ether (MO-TMS) formation, which is disadvantageous in being labor intensive, but permits the ready characterization of structures through relatively easy determination of the number of C=O and C–OH groups. Furthermore, derivatization usually directs the MS fragmentation and a large body of knowledge is available on genesis of specific ions generated within GC/MS analysis. This allows for easier determination of placement of functional groups on the steroid molecule [3]. GC/MS analysis is the superior technology to separate epimeric steroids, so is particularly useful in disorders of metabolism such as 17β-hydroxysteroid dehydrogenase type 3 deficiency, 5α-reductase type 2 deficiency, apparent mineralocorticoid excess (AME) and apparent cortisone reductase deficiency (ACRD).

Using the GC/MS in scan mode allows for non-targeted steroid profiling and for the discovery of novel compounds, metabolomes and pathways of synthesis and metabolism. Scanned data can be stored indefinitely for future revisiting. These advantages make the technique as a “discovery tool”. Table 3 lists several of the circumstances in which GC/MS analysis prevails. Finally, while sufficient specificity in steroid identification can be achieved by a single-stage (e.g. single quadrupole) mass spectrometer, GC allied to tandem mass spectrometers (bench top GC/MS/MS ion-trap instruments) have been long available. In certain circumstances, particularly when small amounts of a targeted analyte are to be measured in the presence of high background, these instruments are invaluable. This is discussed in detail in a paper in this volume [6].

**Table 3:** When GC/MS is preferred technique in steroid analysis.

Clinical practice
1. First-pass investigation of challenging patients- an integrated picture of synthesis and metabolism obtained
2. More reliable diagnosis of metabolism versus synthesis disorders, e.g. AME syndrome, ACRD, 5α-reductase deficiency
3. Defining new metabolomes of biosynthetic and metabolic disorders
4. Less invasive sampling in pediatrics
5. Prenatal diagnosis of steroid biosynthetic disorders

Basic science applications
1. Study of developmentally related synthesis and metabolism changes. Example defining pregnancy “back-door” pathway to active androgens in ORD.
2. Mouse models of human disorders, defining the mouse metabolome and documenting changes in genetically altered animals

Doping in sports
1. Identification of previously unseen designer drugs and metabolites, e.g. tetrahydrogestrinone
2. Stable carbon isotope ratio measurements (in the form of GC/C/MS)

## 4 Specific advantages of GC/MS urine profiling in pediatrics

The sample volume required for steroid hormone analysis employing LC/MS/MS has been significantly reduced compared with immunoassays and steroid hormone profiles have been successfully established as diagnostic tests to differentiate between defects in steroidogenesis. This technology has been proven successful as second-tier or confirmation tests in neonatal congenital adrenal hyperplasia (CAH) screening programs [7], [8] relying on blood spot analysis. However, if used as a confirmation test or as a diagnostic test in patients with later manifestation, one has to bear in mind that this methodology relies on blood sampling, which in a pediatric setting is often traumatizing for patients and parents. Furthermore, blood sampling reflects just a single time point rather than an integrated picture of the steroid metabolome over a longer time span as obtained by GC/MS urine steroid analysis. Applying specific diagnostic metabolite ratios allows for the straightforward diagnosis of conditions of adrenal origin and other disorders of sex development without needing timed sample collection [2], [3], [9], [10].

### 4.1 GC/MS as a discovery tool for novel metabolomes and creating novel hypotheses

#### 4.1.1 The example of P450 oxidoreductase deficiency

The metabolome of what is now termed P450 oxidoreductase deficiency (ORD) was accurately described long ago by thin layer chromatography and GC/MS urine profile analysis and was named combined 17α-hydroxylase/21-hydroxylase deficiency [11], [12], [13]. Patients with ORD present with a pathognomonic urine steroid profile showing increased metabolites typically seen in 21-hydroxylase deficiency, such as pregnanetriol, 17-OH-pregnanolone and pregnanetriolone (17OHP and 21-deoxycortisol metabolites) increased excretion of mineralocorticoid precursors (corticosterone metabolites) and decreased excretion of active androgen metabolites [13]. Interestingly, over the last 30 years there have been multiple publications of misdiagnoses of this condition, usually as variants of 17α-hydroxylase deficiency, because insufficient plasma analytes were measured by conventional techniques to uncover both the apparent 17α- and 21-hydroxylase deficiencies [13]. In 2004, the true cause of this condition was established as deficiency of P450 oxidoreductase, the enzyme essential for providing the electrons required by many steroid hydroxylases – cytochrome P450 type II enzymes [14], [15]. A further paradox is that 46,XX babies are occasionally born with virilized external genitalia, and 46,XY individuals are frequently born with undermasculinized external genitalia. GC/MS provided an ideal technique for diagnosis of the condition as all metabolites of both corticosterone and 17OHP could be analyzed in a single chromatogram [16].

#### 4.1.2 The role of GC/MS in uncovering the cause of genital ambiguity in ORD

**Fig. 2:**
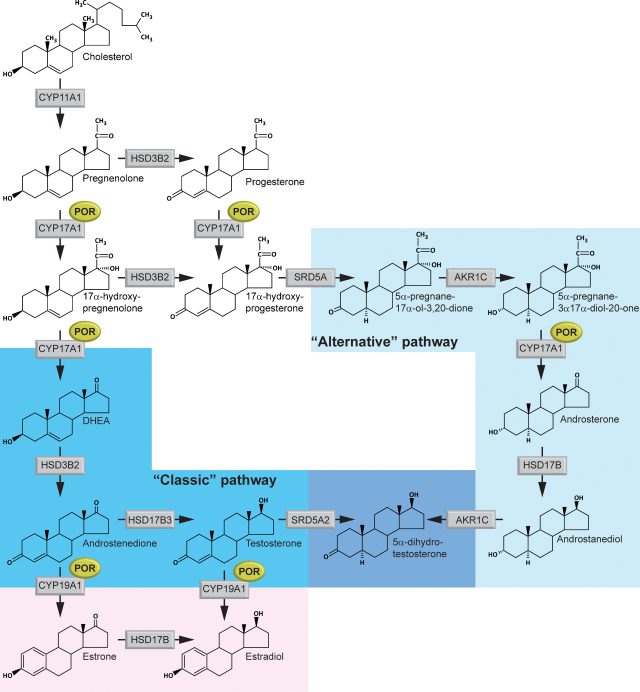
Proposed “back-door” pathway to androgens in ORD pregnancy. Proposed production of dihydrotestosterone via 5α-reduced metabolites 5α-17-OH-pregnanolone and androsterone.

Longitudinal monitoring of pregnancies carrying fetuses with ORD revealed severely decreased estriol synthesis. Maternal signs of virilization by the 23rd week of gestation were also reported, which is often associated with aromatase deficiency. However, pathognomonic urinary metabolite analytes for diagnosis of aromatase deficiency were not significantly increased. An increased excretion of the normally minor metabolite 5α-pregnane-3β,20α-diol (epiallopregnanediol), an epimer of the main progesterone metabolite pregnanediol (5β-pregnane-3α,20α-diol), was detected. Epiallopregnanediol is largely the maternal urinary excretion product of fetal 5-pregnene-3β,20α-diol, itself the principal metabolite of pregnenolone. Since pregnenolone is the precursor of all hormonal steroids, including estriol, this suggested an early block in the estriol biosynthesis pathway in the fetal adrenal [17]. Remarkably, a rapid increase in the androgen metabolite androsterone was noted in the first trimester with a peak at around 20 weeks. This suggested increased synthesis of testosterone and 5α-dihydrotestosterone as a probable cause of the maternal virilization. The clinical picture, together with biochemical evidence gained by urine steroid GC/MS analysis, led to the hypothesis of the existence of an alternative “back-door” pathway of androgen synthesis [15] relying on intermediates similar to a pathway described in the testis of the Tammar Wallaby pouch young [18]. The existence of this “back-door” pathway to androgen synthesis was further supported by analysis of the urine steroid metabolites by GC/MS of additional ORD patients [19] (Fig. 2).

This example highlights the power of GC/MS analysis to characterize new disease entities; to define developmental changes during pregnancy, and to generate novel hypothesis such as the existence of the alternative “back-door” pathway to active androgens. These studies would have been challenging for tandem LC/MS/MS. GC/MS of urine metabolites clearly allows prenatal testing for ORD, as it does for Smith–Lemli–Opitz syndrome (SLOS) [20] and steroid sulfatase deficiency [21]. No LC/MS/MS methods utilizing maternal serum steroids are yet available for these disorders.

### 4.2 The example of apparent cortisone reductase deficiency (hexose-6-phosphate dehydrogenase deficiency)

**Fig. 3:**
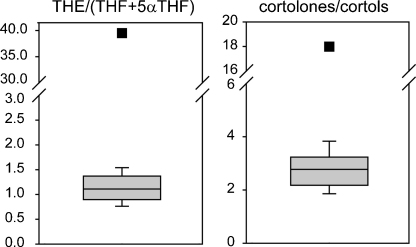
Diagnostic ratios of ACRD. The diagnostic ratios are of major 11-oxo over 11-hydroxy compounds. The normal means, 5th and 95th centiles are given. The square symbols are the ACRD patient results.

As for ORD the history of this disorder goes back 25 years when the unusual metabolome was first indicated [22], [23], [24]. This condition was described in two sisters with hirsutism and increased cortisol production without signs and symptoms of Cushing syndrome. The patients presented with increased excretion of tetrahydrocortisone (THE) and related 11-oxo-steroids relative to 11β-hydroxylated compounds, resulting in the term “apparent cortisone reductase deficiency” (ACRD) being applied to the condition. Several other patients have been described in the last few years and exhaustive studies have shown that defects in hexose-6-phosphate dehydrogenase resulting in a lack of reduced nicotinamide adenine dinucleotide phosphate underlie the condition. GC/MS was essential in diagnosing these patients as well as proving the deficiency through studying the metabolome of the mouse model of the condition [25]. The increased and irreversible conversion of cortisol to cortisone presumably protects against the negative effects of hypercortisolism in this condition. Hyperandrogenism is secondary to increased cortisol clearance and the main clinical manifestation of ACRD. Ratios of cortisol to cortisone metabolites (THE/[THF + 5αTHF], cortolones/cortols) simplify the diagnosis of this condition (Fig. 3).

It would have been almost impossible to define this disorder and elucidate the pathophysiology of ACRD without GC/MS of urinary metabolites. This condition also illustrates the importance of *metabolite* assays in certain instances. The measurement of urinary cortisol and cortisone using LC/MS/MS has been described [26]. However, applying this diagnostic technology would have most likely failed to establish the diagnosis of ACRD, because in patients, the ratio of unmetabolized cortisol to cortisone is normal; thus does not show evidence of apparent 11βHSD deficiency. ACRD is another key example of the power of urine steroid analysis GC/MS employed as a discovery tool in undefined disease entities.

## 5 Improving the presentation of GC/MS data

A major shortcoming of clinical GC/MS urine steroid profiling is in data presentation. Our current standard SIM analysis includes more than 30 steroid hormone metabolites, most of which are only familiar names and structures to the expert. Tabulating only the quantified amounts of steroid metabolites can be confusing and elusive. While naming and measuring these metabolites remains important, improvement in the visual presentation of data has been made.

In steroid profiling, there are two types of data in common use: (1) *quantitative data* of individual metabolites and (2) *diagnostic ratios* of precursor metabolites to product metabolites.

### 5.1 Quantitative data

Quantitative data on excretion of individual steroids is ideal but requires accurate 24 h sampling. Most steroid disorders can be identified from “spot” or random samples but in certain cases, for example when evaluating aldosterone synthesis, accurate quantification is essential.

**Fig. 4:**
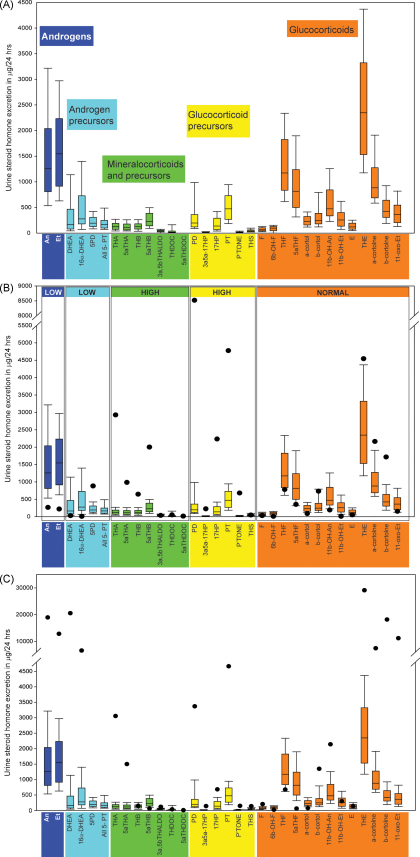
Report of urinary steroid quantitation as μg/24 h. The upper panel shows the normal excretion of metabolites in adults. The color-coding is the same as described in Fig. 1. Mean values, 5th and 95th percentile and given. The middle panel shows results quantified in the 24-h urine of a patient, with individual measurement results illustrated by black dots. This case was diagnosed as ORD because of the high excretion of metabolites of pregnenolone (highlighted in light blue) and progesterone (yellow) with concurrent elevation of corticosterone pathway metabolites (green). The lower panel shows results for an ACRD patient. Note the high excretion of androgen metabolites and the high excretion of cortisol metabolites containing an 11-carbonyl (e.g. THE, cortolones).

The pathway to steroid hormone production is commonly divided into the synthesis of mineralocorticoid, glucocorticoids and sex steroids (Fig. 1). We have added two groups to this classification; ‘androgen precursors’ includes the Δ^5^-steroids pregnenolone, 17OH-pregenenolone, DHEA and the Δ^4^-steroids androstenedione and their metabolites as this represent the main pathway to active androgens. The second additional group is of ‘glucocorticoid precursors’, which consists of the Δ^4^-steroids progesterone, 17OH-progesterone and 21-deoxycortisol and their metabolites (Fig. 1). We have used our normal controls to develop normal ranges in groupings for metabolites of androgens, androgen precursors, mineralocorticoids and precursors, glucocorticoid precursors and glucocorticoids (Fig. 4A). The GC/MS profile of an individual patient is plotted for each determined parameter versus the normal reference population. Such profiles allow for an immediate overview on a rather complex constellation. As exemplified in Fig. 4B, it is obvious that the individual has a condition with androgen deficiency involving androgen precursors, in combination with increased mineralocorticoid precursors and increased amounts of glucocorticoid precursors, but normal glucocorticoids under non-stress condition, the hallmarks of ORD.

### 5.2 Diagnostic ratios

Most hormonal imbalances caused by enzyme deficiencies or “blocks” cause depletion of a steroid product and build-up of the upstream precursors. Thus, a ratio of metabolites of the product to metabolites of the product should indicate if there is such a block. Such ratios have been used for years and are available for most steroid disorders (Table 4). Although this approach simplifies data presentation and does not require timed samples, most clinical “end users” are often alienated by such numerical data presentation and only an exclusive audience with a special interest in steroid hormone metabolism gets benefit from such results with others relying on result interpretation by a few experts. To simplify this problem and make this important information available to a broader clinical audience, we have developed disease specific graphics based on substrate/product ratios. Certainly some knowledge on steroid metabolites is required, but graphical presentation of urine steroid profiles as quantitative reports versus normal controls and diagnostic “precursor metabolite/product metabolite” ratios will allow for wider use of these data sets.

**Table 4:** Diagnostic ratios for differential diagnosis of inborn errors of steroidogenesis and steroid metabolism.

Enzyme defect (*Gene*)	Diagnostic ratio
21-Hydroxylase deficiency (*CYP21A2*)	17HP/(THE + THF + 5αTHF)
	PT/(THE + THF + 5αTHF)
	PTONE/(THE + THF + 5αTHF

11β-Hydroxylase deficiency (*CYP11B1*)	THS/(THE + THF + 5αTHF)
	
17α-Hydroxylase deficiency (*CYP17A1*)	(THA + 5αTHA + THB + 5αTHB)/(THE + THF + 5αTHF)
	(THA + 5αTHA + THB + 5αTHB)/(An + Et)

3β-Hydroxysteroid dehydrogenase deficiency (*HSD3B2*)	DHEA/(THE + THF + 5αTHF)
	5PT/(THE + THF + 5αTHF)

P450 oxidoreductase deficiency (ORD) (*POR*)	(17HP + PT)/(An + Et)
	(17HP + PT)/(THE + THF + 5αTHF)
	PD/(THE + THF + 5αTHF)
	5PD/(THE + THF + 5αTHF)
	(THA + 5αTHA + THB + 5αTHB/(THE + THF + 5αTHF)

Apparent mineralocorticoid excess (AME) (*HSD11B2*)	F/E
	(THF + 5αTHF)/THE
	Cortols/cortolones
	(F + E)/(THF + 5αTHF + THE)

Apparent cortisone reductase deficiency (ACRD) (*H6PDH*)	THE/(THF + 5αTHF)
	Cortolones/cortols

5α-Reductase deficiency (*SRD5A2*)	Et/An
	THB/5αTHB
	THF/5αTHF

17β-Hydroxysteroid dehydrogenase deficiency (*HSD17B3*)	(An + Et)/(THE + THF + 5αTHF)

**Fig. 5:**
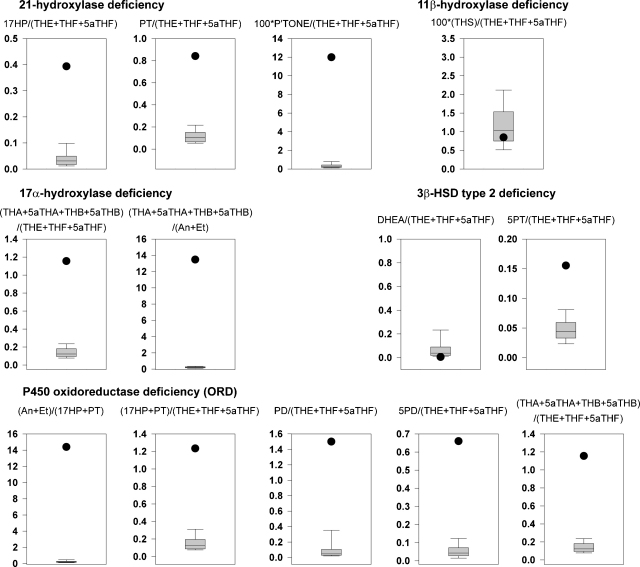
Diagnostic ratio panel for CAH. Quantitative data (black dot) for the ORD patient compared to normative data appropriate for several forms of CAH. It is clear that by using the designated ratios that the diagnosis can be accurately given. Only the ORD ratios are all abnormal.

The CAH panel contains three specific ratios for 21-hydroxylase deficiency, one for 11β-hydroxylase deficiency, two for 17α-hydroxylase deficiency and 3β-HSD type 2 deficiency each, and five for ORD (Fig. 5). Applying these representations leads to immediate differentiation between different forms of known errors in steroidogenesis. Patients’ profiles and ratio combinations not fitting these established diagnostic criteria, thus excluding established conditions, can be reanalyzed by various techniques to elucidate novel underlying defects.

## 6 Conclusions

With the advent of high throughput LC/MS/MS most classical steroid analytic techniques can be replaced by measurement of hormones and precursors; in particular for more common conditions and well-known analytes. Accurate values are generally being obtained for the first time, particularly in female and pediatric patients with low concentrations of androgens and estrogens although work is still necessary in improving reproducibility between laboratories. In spite of this advance, GC/MS will continuously play an active role in studying rare and undefined conditions and retains its place as the pre-eminent discovery tool for defining new and aberrant metabolic pathways and for first-time characterization of unknown steroids. It remains the best technique for studying “metabolic” in contrast to “biosynthetic” disorders (e.g. 5α-reductase deficiency, AME, ACRD), and is good in pediatric situations when it is desirable to minimize blood sampling. The technique is still at the core of methods for prenatal diagnosis of steroid disorders by analysis of maternal urine or amniotic fluid. Thus, we regard both mass spectrometric techniques as complementary rather than competing technologies.

## References

[1] Soldin S.J., Soldin O.P. (2009). Steroid hormone analysis by tandem mass spectrometry. Clin. Chem..

[2] Shackleton C.H.L., Marcos P.G.M., Gross M., Caprioli R. (2006). The Encyclopedia of Mass Spectrometry.

[3] Shackleton C.H., Blau N., Duren M., Gibson K.M. (2008). Laboratory Guide to the Methods in Biochemical Genetics.

[4] Shackleton C.H. (1986). Profiling steroid hormones and urinary steroids. J. Chromatogr..

[5] Griffiths W.J., Shackleton C.H.L., Sjövall J., Caprioli R. (2006). The Encyclopedia of Mass Spectrometry.

[6] C.H. Shackleton, Clinical steroid mass spectrometry: a 45-year history culminating in HPLC MS/MS becoming an essential tool for patient diagnosis, J. Steroid. Biochem. Mol. Biol., in press. 10.1016/j.jsbmb.2010.02.017 20188832

[7] Minutti C.Z., Lacey J.M., Magera M.J., Hahn S.H., McCann M., Schulze A., Cheillan D., Dorche C., Chace D.H., Lymp J.F., Zimmerman D., Rinaldo P., Matern D. (2004). Steroid profiling by tandem mass spectrometry improves the positive predictive value of newborn screening for congenital adrenal hyperplasia. J. Clin. Endocrinol. Metab..

[8] Janzen N., Peter M., Sander S., Steuerwald U., Terhardt M., Holtkamp U., Sander J. (2007). Newborn screening for congenital adrenal hyperplasia: additional steroid profile using liquid chromatography–tandem mass spectrometry. J. Clin. Endocrinol. Metab..

[9] Caulfield M.P., Lynn T., Gottschalk M.E., Jones K.L., Taylor N.F., Malunowicz E.M., Shackleton C.H.L., Reitz R.E., Fisher D.A. (2002). The diagnosis of congenital adrenal hyperplasia in the newborn by gas chromatography/mass spectrometry analysis of random urine specimens. J. Clin. Endocrinol. Metab..

[10] Wudy S.A., Hartmann M., Homoki J. (2000). Hormonal diagnosis of 21-hydroxylase deficiency in plasma and urine of neonates using benchtop gas chromatography–mass spectrometry. J. Endocrinol..

[11] Shackleton C.H., Taylor N.F., Honour J.W. (1980).

[12] Peterson R.E., Imperato-McGinley J., Gautier T., Shackleton C. (1985). Male pseudohermaphroditism due to multiple defects in steroid-biosynthetic microsomal mixed-function oxidases. A new variant of congenital adrenal hyperplasia. N. Engl. J. Med..

[13] Shackleton C., Malunowicz E. (2003). Apparent pregnene hydroxylation deficiency (APHD): seeking the parentage of an orphan metabolome. Steroids.

[14] Fluck C.E., Tajima T., Pandey A.V., Arlt W., Okuhara K., Verge C.F., Jabs E.W., Mendonca B.B., Fujieda K., Miller W.L. (2004). Mutant P450 oxidoreductase causes disordered steroidogenesis with and without Antley-Bixler syndrome. Nat. Genet..

[15] Arlt W., Walker E.A., Draper N., Ivison H.E., Ride J.P., Hammer F., Chalder S.M., Borucka-Mankiewicz M., Hauffa B.P., Malunowicz E.M., Stewart P.M., Shackleton C.H. (2004). Congenital adrenal hyperplasia caused by mutant P450 oxidoreductase and human androgen synthesis: analytical study. Lancet.

[16] Shackleton C., Marcos J., Malunowicz E.M., Szarras-Czapnik M., Jira P., Taylor N.F., Murphy N., Crushell E., Gottschalk M., Hauffa B., Cragun D.L., Hopkin R.J., Adachi M., Arlt W. (2004). Biochemical diagnosis of Antley-Bixler syndrome by steroid analysis. Am. J. Med. Genet..

[17] Shackleton C., Marcos J., Arlt W., Hauffa B.P. (2004). Prenatal diagnosis of P450 oxidoreductase deficiency (ORD): a disorder causing low pregnancy estriol, maternal and fetal virilization, and the Antley-Bixler syndrome phenotype. Am. J. Med. Genet..

[18] Wilson J.D, Auchus R.J., Leihy M.W., Guryev O.L., Estabrook R.W., Osborn S.M., Shaw G., Renfree M.B. (2003). 5α-Androstane-3α,17β-diol is formed in Tammar Wallaby Pouch young testes by a pathway involving 5α-pregnane-3α,17α-diol-20-one as a key intermediate. Endocrinology.

[19] Homma K., Hasegawa T., Nagai T., Adachi M., Horikawa R., Fujiwara I., Tajima T., Takeda R., Fukami M., Ogata T. (2006). Urine steroid hormone profile analysis in cytochrome P450 oxidoreductase deficiency: implication for the backdoor pathway to dihydrotestosterone. J. Clin. Endocrinol. Metab..

[20] Shackleton C.H., Marcos J., Palomaki G.E., Craig W.Y., Kelley R.I., Kratz L.E., Haddow J.E. (2007). Dehydrosteroid measurements in maternal urine or serum for the prenatal diagnosis of Smith–Lemli–Opitz syndrome (SLOS). Am. J. Med. Genet..

[21] Marcos J., Craig W.Y., Palomaki G.E., Kloza E.M., Haddow J.E., Roberson M., Bradley L.A., Shackleton C.H. (2009). Maternal urine and serum steroid measurements to identify steroid sulfatase deficiency (STSD) in second trimester pregnancies. Prenat. Diagn..

[22] Taylor N.F., Barlett W.A., Dawson D.J. (1984). Cortisone reductase deficiency: evidence for a new inborn error of metabolism. J. Endocrinol..

[23] Phillipou G., Higgins B.A. (1985). A new defect in the peripheral conversion of cortisone to cortisol. J. Steroid Biochem..

[24] Phillipov G., Palermo M., Shackleton C.H. (1996). Apparent cortisone reductase deficiency: a unique form of hypercortisolism. J. Clin. Endocrinol. Metab..

[25] Lavery G.G., Walker E.A., Tiganescu A., Ride J.P., Shackleton C.H., Tomlinson J.W., Connell J.M., Ray D.W., Biason-Lauber A., Malunowicz E.M., Arlt W., Stewart P.M. (2008). Steroid biomarkers and genetic studies reveal inactivating mutations in hexose-6-phosphate dehydrogenase in patients with cortisone reductase deficiency. J. Clin. Endocrinol. Metab..

[26] Taylor R.L., Machacek D., Singh R.J. (2002). Validation of a high-throughput liquid chromatography–tandem mass spectrometry method for urinary cortisol and cortisone. Clin. Chem..

